# Magnetic gastrointestinal anastomosis technique for the treatment of duodenal stricture: the first clinical report

**DOI:** 10.1055/a-2612-3706

**Published:** 2025-06-26

**Authors:** Jiyu Zhang, Miao Shi, Qingfen Zheng, Lili Wang, Lixia Zhao, Dan Liu, Bingrong Liu

**Affiliations:** 1 191599 Department of Gastroenterology and Hepatology, The First Affiliated Hospital of Zhengzhou University, Zhengzhou, China

In this report, we present a successful gastrointestinal anastomosis performed using the magnetic compression anastomosis technique in a patient with duodenal stricture.

**Fig. 1:**
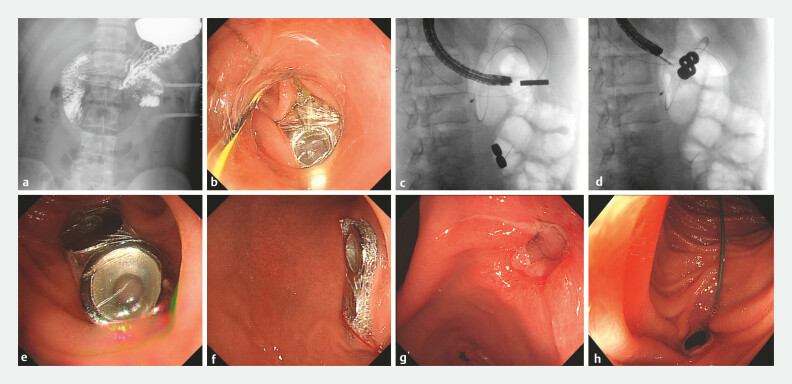
Radiographic and endoscopic images. **a** Preoperative upper gastrointestinal tract radiographic image showing duodenal stasis. **b** A magnetic device was placed within the intestine. **c** Under X-ray guidance, the position of the first magnetic device was adjusted by pulling the thread, facilitating its attraction to the second device. **d** Abdominal X-ray showing the two bonded magnetic devices, achieving gastric and intestinal adhesion. **e** Endoscopic image showing one magnetic device positioned on the intestine wall. **f** Endoscopic image showing one magnetic device positioned on the posterior gastric wall. **g, h** Postoperative follow-up endoscopy demonstrating a transitable anastomosis.

A 22-year-old man experienced repeated abdominal distension and recurrent vomiting for 3 months. Upper gastrointestinal tract radiography and endoscopic examination confirmed the presence of duodenal stasis ( Fig. 1 **a** ), which was suspected to be caused by superior mesenteric artery syndrome. Conservative measures failed to alleviate the patient’s symptoms, and he declined surgical intervention. Consequently, we opted to perform a gastrointestinal anastomosis by the magnetic compression anastomosis technique.

To create the anastomosis, we prepared four ring-shaped magnets and assembled them into two figure-of-eight magnetic devices (20 mm in length and 10 mm in width), with one of the devices connected by a thread. During the procedure, one magnetic device was delivered past the duodenal stricture using biopsy forceps and positioned in the intestine, accompanied by a contrast guidewire to indicate the precise location of the magnet ( Fig. 1 **b** ). Subsequently, the second magnetic device was delivered into the stomach ( Fig. 1 **c** ). Under X-ray guidance, the position of the first magnetic device was adjusted by pulling the thread, facilitating its attraction to the second device ( Fig. 1 **d** ). Finally, the guidewire was withdrawn, and the thread was cut off and removed, with endoscopic visualization confirming that one magnet remained in the intestine and the other in the gastric body ( Fig. 1 **e, f** ).

The magnetic devices induced necrosis at the compression site, promoting tissue adhesion and subsequent anastomosis formation. At 1 week post-procedure, the patient’s symptoms had alleviated, and 3 weeks later, the anastomosis was accessible via endoscopy without complications ( Fig. 1 **g, h** ).

**Fig. 2:**
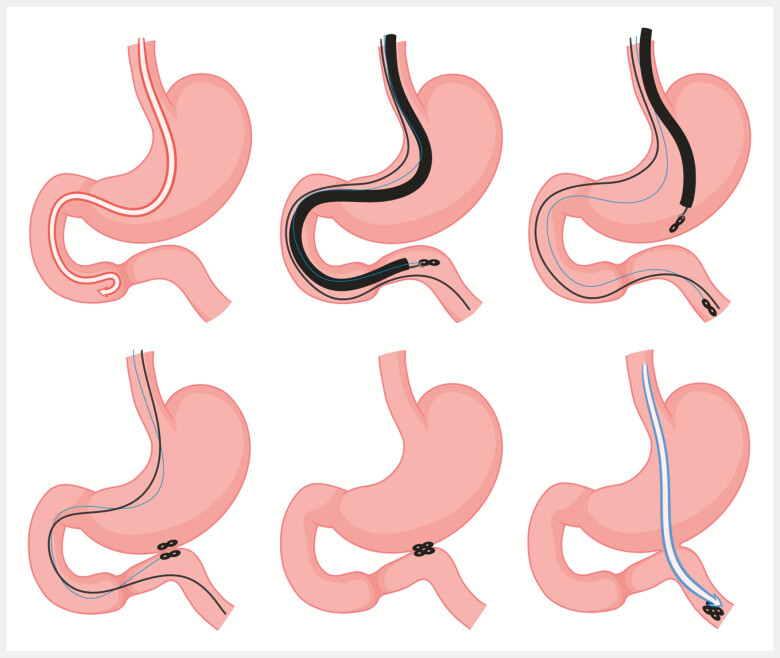
Schematic diagram of the procedure.

This case illustrates the efficacy of a novel, less invasive gastrointestinal anastomosis technique ( Fig. 2 , Video 1 ); the magnet-assisted approach demonstrated successful endoscopic treatment for duodenal strictures.

Magnetic gastrointestinal anastomosis technique for the treatment of duodenal stricture.Video 1

Endoscopy_UCTN_Code_TTT_1AT_2AD

